# Genetic diversity of hepatitis E virus (HEV) strains derived from humans, swine and wild boars in Croatia from 2010 to 2017

**DOI:** 10.1186/s12879-019-3906-6

**Published:** 2019-03-19

**Authors:** Lorena Jemeršić, Jelena Prpić, Dragan Brnić, Tomislav Keros, Nenad Pandak, Oktavija Đaković Rode

**Affiliations:** 1 Croatian Veterinary Institute, Savska cesta 143, 10 000 Zagreb, Croatia; 2 General Hospital “Josip Bencevic”, University of Osijek, Faculty of Medicine, Andrije Stampara 42, 35000 Slavonski Brod, Croatia; 3 University Hospital for Infectious Diseases “Dr. Fran Mihaljevic”, Mirogojska 8, 10 000 Zagreb, Croatia; 4 University of Zagreb School of Dental Medicine, Gundulićeva 5, 10000 Zagreb, Croatia

**Keywords:** Hepatitis E virus genotyping, Human, Swine, Wild boar, Croatia

## Abstract

**Background:**

To fulfill epidemiological data and investigate possible interspecies transmission, this study shall attempt to sequence representative HEV strains of human, swine and wild boar origin collected from 2010 to 2017 in Croatia.

**Methods:**

In total, 174 anti-HEV antibody positive human sera samples; 1419 blood or faeces samples of swine, as well as 720 tissue and/or blood samples of wild boar originating from different counties (18 in total) in Croatia were tested for the presence of HEV RNA.

**Results:**

HEV RNA was detected in 26 human sera samples (14.9%; 95% CI 10.4–21.0%). HEV RNA was detected in 216 tested swine (15.2%; 95% CI 13.5–17.1%), regardless of age, farm breeding system or geographical origin. Viral RNA was also detectable in faeces samples which prove that swine actively participate in shedding HEV into the environment. Of the total of 720 tested wild boar samples, 83 were HEV RNA positive (11.5, 95% CI 9.4–14.1%) originating from six counties. According to the sequence analysis all strains have shown to be members of *Orthohepevirus A* genotype HEV-3, regardless of host. The genotyping results confirm grouping of sequences into four subtypes of HEV strains of which subtypes 3a and 3c belong to the general cluster 3abchij, and were predominately detected during the study, while subtypes 3e and 3f fall within cluster 3efg. Strains within subtypes 3a and 3e were found in humans, swine and wild boars; subtype 3c strains were derived from humans and swine, whereas subtype 3f strains were found only in humans. Strains belonging to subtypes 3a and 3c were derived during the entire investigated period and may be considered endemic in Croatia, whereas strains within subtypes 3e and 3f were detected sporadically indicating the possibility of newly imported infections.

**Conclusions:**

All detected strains show to be genetically highly related to strains found in humans and/or animals from other European Countries, indicating that trade of live animals or wild boar movement increases the risk of HEV infection spread. Furthermore, homologous strains found in different investigated species within this study indicate interspecies transmission of HEV and/or an existence of an accessible mutual source of infection.

**Electronic supplementary material:**

The online version of this article (10.1186/s12879-019-3906-6) contains supplementary material, which is available to authorized users.

## Background

Hepatitis E is an enterically transmitted, mostly self limiting and waterborne acute viral infection in developing countries of Asia, Africa and Central America. In Europe and North America autochthonous infections are sporadic and no cases of HEV transmission through contaminated water have been reported [1–4]. Even so, HEV RNA was derived from river water [5] therefore it may potentially be a source of infection. Conversely, bottled water is not considered a risk factor for HEV infection since a significantly lower rate of anti-HEV IgG antibodies was found in people that consumed bottled water when compared to the overall IgG seroprevalence [6].

The significance of the disease as a public health problem is arising especially as extra-hepatic manifestations associated with hepatitis E infection [7], as well as cases of chronic, even fulminate infections in immunocompromised patients are recorded [1, 8–10]. The causative agent, the hepatitis E virus (HEV) is a small (27–34 nm), non-enveloped single-stranded virus with a linear positive-sense RNA genome of approximately 7.2 kb in length [11]. According to its genetic diversity, HEV classifies into the *Hepeviridae family* consisting of two genera, *Orthohepevirus (A, B, C* and *D)* and *Piscihepevirus* [12]. Members of *Orthohepevirus A* species are currently divided into seven genotypes [13]. Recently, an eighth genotype has been proposed [14]. HEV-1 and HEV-2 are predominately found in developing countries and are human specific; HEV-3 is distributed worldwide and has been derived from both humans and animals, whereas HEV-4, that also shows a zoonotic potential, is considered to be indigenous to Asia [15]. Even so, cases of infection by HEV-4 have recently also been reported in Europe [16]. Novel genotypes of HEV as well as hosts are consecutively being recognised, however from the epidemiological point of view, only members of the *Suidae* family are still considered as the only true reservoirs of HEV. Due to the fact that HEV infection remains asymptomatic in animal mammals, therefore in swine as well, and due to a long incubation period reported in patients, only a limited number of human infections are directly linked to the consumption of food of animal origin as the source of infection [17–19]. Therefore, the comparison of sequences of HEV RNA derived from humans and swine remains the only method of choice for detecting possible routes of transmission [20].

Since 2009 HEV RNA has been continuously detected in swine and wild boars in Croatia [21, 22], and has been followed by the report of the first human case of autochthonous hepatitis E in 2012 [23]. Since then, sporadically infected patients showing signs of hepatic disorders and positive for anti-HEV antibodies and for HEV RNA have regularly been recorded [24]. The seroprevalence in non A-C hepatitis patients has shown to be 10.7% and in HIV positive individuals 1.1% [24]. Likewise, a pilot study revealed a seroprevalence of 5.6% in asymptomatic individuals [25]. A high seroprevalence of 32.9 and 31.1% was detected in swine and wild boar in Croatia, respectively [22]. However, the relation among the reported cases in humans, swine and wild boars has not been investigated. Therefore, this study presents the first results of an epidemiological observation conducted using samples collected between 2010 and 2017 in Croatia based on genotyping and phylogenetic comparison of HEV strains derived from infected humans, swine and wild boars with an aim to reveal potential interspecies transmission or identify mutual sources of infection.

## Methods

### Sampling

#### Human samples

Sera samples (174 in total) originating from anti-HEV antibody positive patients were collected from 2012 to 2017 from different regions in Croatia by the National Referent Centre for viral hepatitis infections at the University Hospital for Infectious Diseases “Dr. Fran Mihaljevic” in Zagreb in accordance with ethical principles. The panel also included 12 archive samples of patient with hepatic lesions of unknown origin. All tested patients (age range = 16 to 90; median age = 43.6; gender proportion = 1.07:1 of female versus male positive patients) demonstrated signs of hepatic lesions and were negative for hepatitis A, B and C and were tested for the presence of HEV according to the National guidelines for diagnosis and treatment of hepatitis E in Croatia [24]. Prior to RNA testing the presence of specific IgM and/or IgG anti-HEV antibodies was confirmed by a commercially available enzyme immunoassay (EIA; *recom*Well HEV IgG, IgM; Mikrogen GmbH, Neuried, Germany) and a line immunoassay (LIA; *recom*Line HEV IgG/IgM, Mikrogen GmbH, Neuried, Germany). The samples were stored at -80 °C until tested at the Croatian veterinary institute in Zagreb by RT-PCR.

#### Swine and wild boar samples

Swine (in total 1419) and wild boar (in total 720) samples were routinely collected in accordance with an ongoing national classical swine fever monitoring program and a program for the detection of the distribution of hepatitis E virus in swine and wild boars in the Republic of Croatia.

Samples were randomly chosen taking into account the sample quality, age/breeding categories and geographical distribution of swine and wild boars (based on the estimated population size per County, with a 5 or 10% prevalence and a 95% or 90% probability). The samples consisted of blood (collected by venepuncture of *v. Jugularis*) or faeces of swine, as well as blood (collected directly from the heart cavities) and/or spleen, kidney, hepatic and tonsil tissue of shot wild boars during hunting seasons. Tissue samples (100 mg of each) were homogenized by using the FastPrep-24 machine, model 6004–500 (MP Biomedicals, Santa Ana, California, USA) and lysing matrix A, D, and for tonsils matrix M, with addition of 1 ml sterile phosphate buffered saline (PBS). The supernatants were decanted into sterile test tubes and stored at -80 °C until further testing.

Faeces of swine were collected in ten investigated counties from ten large breeding farms with high biosecurity measures and eleven small farms (up to 5 sows) with an open farm breeding system. Faecal samples were pooled (103 pooled samples) on the basis of same origin (farm) and age/breeding category. Faecal samples were resuspended in phosphate-buffered saline (PBS; pH 7.4) in order to obtain 20% *w*/*v* faecal suspensions which were then vortexed for 1 min and centrifuged for 15 min at 1000 g. Supernatants were further centrifuged for 3 min at 18000 g and stored at -80 °C until further testing.

### Study details

**Fig. 1:**
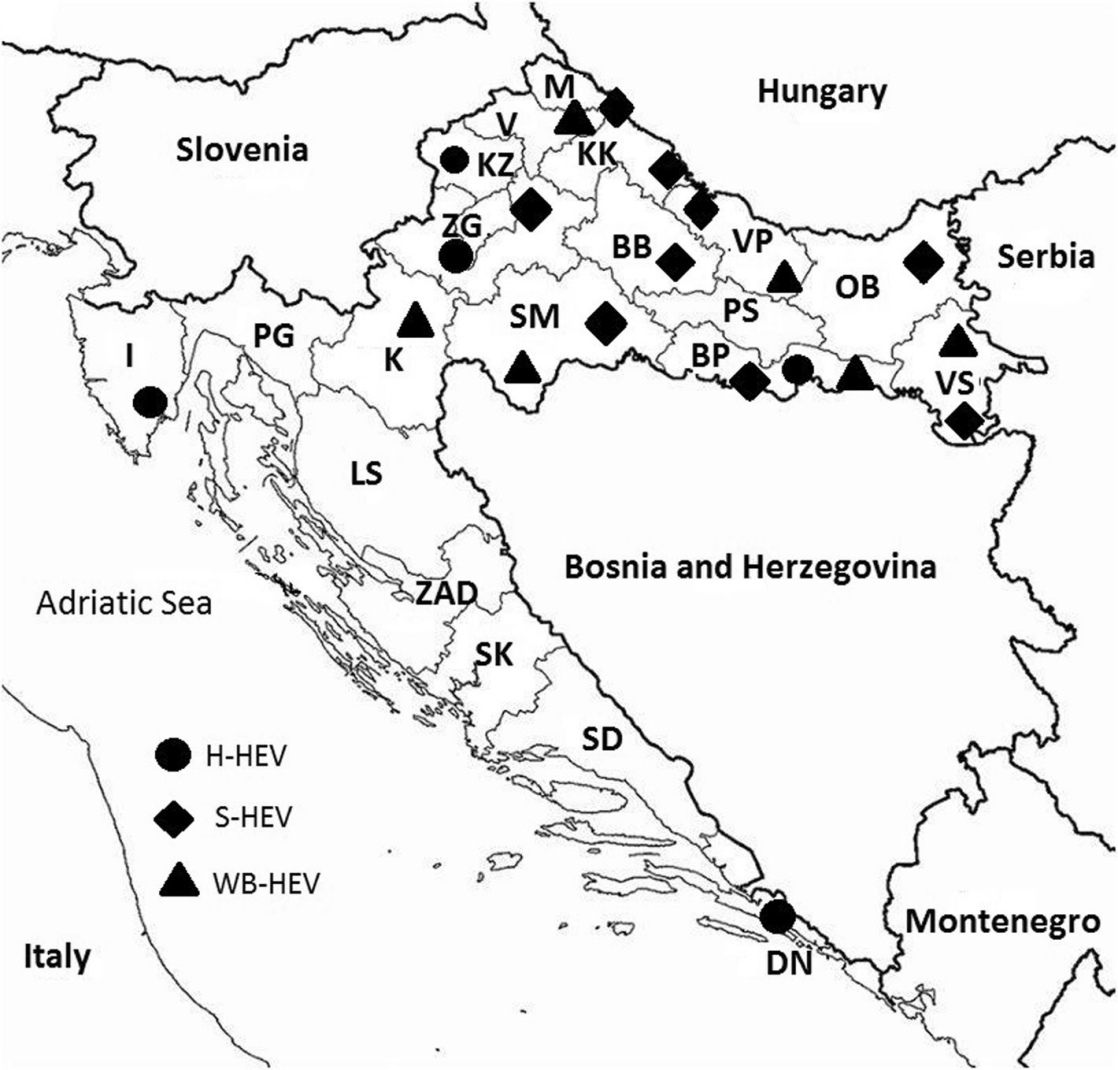
Map of Croatia showing positive RT-PCR findings of HEV in humans (●H-HEV), swine (◆S-HEV) and wild boars (▲WB-HEV) from 2010 to 2017

All geographical data was County level based, where North Croatia included Bjelovar Bilogora (BB), Brod Posavina (BP), Karlovac (K), Koprivnica Krizevci (KK), Krapina Zagorje (KZ), Lika Senj (LS), Medimurje (M), Osijek Baranja (OB), Požega Slavonia (PS), Sisak Moslavina (SM), Virovitica Podravina (VP), Vukovar Srijem (VS) and Zagreb (ZG) counties, and South Croatia included Dubrovnik Neretva (DN), Istria (I), Primorje Gorski kotar (PG), Split Dalmatia (SD), Sibenik Knin (SK), Varazdin (V) and Zadar (ZAD) County that follow the coast of the Adriatic Sea (Fig. 1).

#### RNA extraction and reverse transcription

Viral RNA extraction was conducted by QIAamp viral RNA Mini Kit® (Qiagen, Hilden, Germany) from 140 μl of previously prepared samples according to the producer’s instructions. For nested PCR reverse transcription was performed (GoScript Reverse Transcription System® for RT-PCR, Promega, Madison, Wisconsin, USA) in a GeneAmp PCR System 9700 machine (Applied Biosystems, CarlSGad, California, USA).

#### PCR protocols and sequencing

To identify HEV RNA carriers a real time RT-PCR protocol [26] for detecting a highly conserved fragment within ORF3 was carried out. The amplification was done in a Rotor-Gene Q machine (Qiagen, Hilden, Germany) by the use of commercially available kits (Rotor-Gene Probe RT-PCR kit, Qiagen, Hilden, Germany) according to the producer’s instructions. All positive samples were re-tested by a nested RT-PCR protocol [27] for detecting a variable fragment within ORF1 (242 nt), adequate for sequencing and genotyping [14]. The amplification procedure for the nested PCR (GoTaq® DNA Polymerase kit, Promega, Madison, Wisconsin, USA) was carried out as follows: denaturation at 94 °C for 3 min; incubation at 94 °C for 45 s, 55 °C for 45 s, and 72 °C for 45 s (35 cycles); and incubation at 72 °C for 20 min. PCR products were separated by agarose gel electrophoresis in 1.5% agarose gel stained with a fluorescent dye- (GelStar™ Nucleic Acid Gel Stain 10,000X, Lonza, Walkersville, Maryland, USA) and visualized by UV transillumination. As a positive control, sequences of previously detected fragments of HEV RNA genotype 3 were used (derived from positive swine sera). Negative controls were aliquots of ultra pure water.

All samples (in total 52) containing HEV RNA detectable in a lower cycle threshold value of 25 were sequenced. Seventeen of the tested samples originated from seropositive, probably chronically infected wild boars [22].

PCR products were purified (Wizard® SV gel and PCR Clean up system, Promega, Madison, Wisconsin, USA) and sequenced by Macrogen Inc. (Amsterdam, The Netherlands). Phylogenetic grouping and clustering of all obtained sequences was based on comparison with strains retrieved from the GenBank (Additional file 1), using algorithm BLAST (http://www.ncbi.nlm.nih.gov). Sequences were aligned by ClustalW 1.6 and analyzed using MEGA 5, based on published recommendations [13], whereas the trees were generated using the neighbour-joining method applying the Kimura 2-parameter evolutionary model.

### Statistics

The lower and upper limits of the 95% confidence interval (CI) for a proportion were calculated. To define the differences in the occurrence of hepatitis E in Croatia in domestic pigs and wild boars, Chi-square test was used. *P* ≤ 0.05 was considered statistically significant.

## Results

### PCR results

From the total number (174) of tested human samples that have shown to be anti-HEV antibody positive, 26 (14.9%; 95% CI 10.4–21.0%) of them, were HEV RNA positive, including an archive sample from 2010. HEV RNA was recovered from sera samples only in IgM or IgM/IgG positive patients. On the other hand, all solely IgG anti-HEV antibody positive human samples were negative for the presence of HEV RNA. Positive patients originated from five counties (ZG, PG, KZ, BP and DN) included within this study (Fig. 1).

In swine, HEV RNA was detected in 216 samples (15.2%; 95% CI 13.5–17.1%) in nine investigated counties (BB, BP, KK, M, OB, SM, VP, VS, and ZG). Positive swine faecal samples were found in six counties (BP, M, OB, SK, VS, ZG) regardless of the breeding conditions and age/breeding categories of swine.

HEV RNA was detected in 83 (11.5%; 95% CI 9.4–14.1%) wild boars originating from six counties (BP, K, M, SM, VP and VS) (Fig. 1). All positive wild boars were hunted in regions with a high density of intensive or extensive swine breeding farms (SM, VS and VP).

The highest number of HEV RNA positive/tested swine samples was found in SM (37.4%; 95% CI 30.0–45.5%), VS (20.6%; 95%CI 15.1–27.4%) and OB (18.8%; 95%CI 15.2–23.0%) counties, whereas in wild boars the highest RNA positivity was found in BP (23.8%; 95%CI 17.6–31.4%) and SM (22%; 95%CI 15.8–29.5%) counties. A respectful percentage (15.4%; 95%CI 11.4–20.4%) of positive wild boars was also found in VP County, however this may be due to a limited number of collected samples.

The distribution of all positive samples regardless of host is statistically significantly higher (*p* < 0.05) in Northern Croatia when compared to the South regions following the Adriatic Coast. In numbers, 22 out of 26 (84.6%; 95%CI 66.5–93.9%) positive patients; 215 out of 216 (99.5%; 95%CI 97.4–99.9%) positive swine and all (83) positive wild boars were from the North of Croatia. In total, out of 325 positive cases including all three species, 320 (98.5%; 95%CI 96.5–99.3%) were detected in the North of Croatia. From the South, three patients were from DN and one from I County, while one positive swine was detected in SK County.

### Genotyping results

**Fig. 2:**
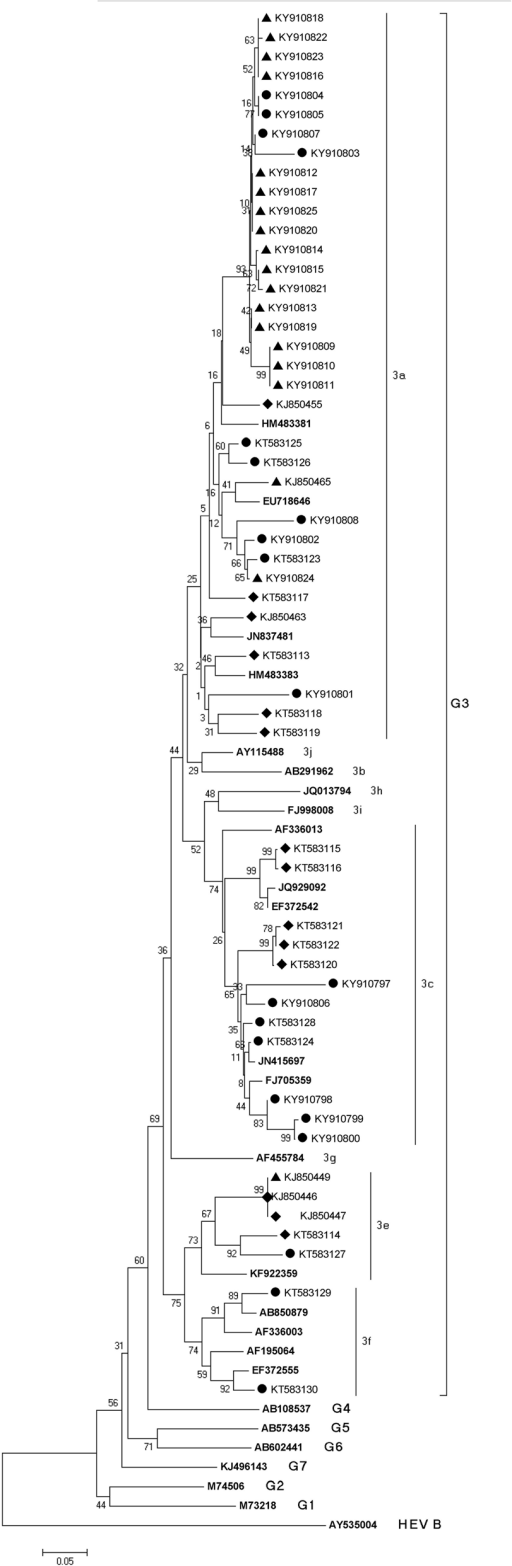
Neighbor-joining phylogenetic tree obtained by the analysis of the partial ORF1 region of HEV strains derived from human (circle symbol), swine (diamond symbol) and wild boar (triangle symbol) samples in Croatia. Genetic distances were calculated using the Kimura two-parameter method. Bootstrap values are presented next to tree nodes. The bar represents 0.05 nucleotide substitution per site

HEV strains derived from humans (H-HEV), swine (S-HEV) and wild boars (WB-HEV) in Croatia from 2010 to 2017 are identified as members of species *Orthohepevirus A*, genotype 3 (Fig. 2). All strains are genetically closely related even though they cluster into two general subgroups defined by [13] and four subtypes (ST) in humans (3a, 3c, 3e and 3f), three in swine (3a, 3c and 3e) and two (3a and 3e) in wild boars (Fig. 2). The majority of HEV strains derived in Croatia belong to subtypes 3a (65.4%; 95%CI 51.8–76.9%) and 3c (23.1%; 95%CI 13.7–36.1%), while strains of subtypes 3a and 3e were detected in all three investigated species. The sequences mostly cluster in regards to the year of detection, not host or county of appearance.

In 2010 strains from swine and wild boar belong to subtypes 3a and 3e and show to be highly genetically related. Only two human sequences, one from 2010 and the other from 2012 belonged to subtype 3f (clade 3efg).

Three sequences were analyzed in 2012 and all of them were derived from human patients. One (KT58323) is strongly genetically related to a H-HEV strain from 2013 (KT583126) and a WB-HEV strain from 2010 (KJ850455); one of them (KT583123) to a H-HEV strain from 2017 (KY910802) and WB-HEV strain (KY910824) from 2016 and the third (KT583124) is highly related to a H-HEV from 2013 from the same County (KT583128) as well as to some S-HEV strains (KT583121, 122 and 120).

Sequences from 2013 are distributed into subtypes 3a, 3c (clade abchij) and 3e (clade efg), regardless of host and are show highly homologous regardless of geographical origin (KT583128 with KT583121, 122 and 120). Only two strongly related sequences (KT583144 and KT583127) had the same geographical origin (Zagreb County).

During 2014, five positive HEV RNA human samples were detected. Three representative ones are shown within the tree and are members of subtype 3a (KY910803 and KY910801) and subtype 3c (KY910806).

In 2015, two sequences of H-HEV samples were identified. They both cluster in subtype 3a and show to be identical to WB-HEV and H-HEV samples from 2014, 2016 and 2017.

Generally, the 2016 and 2017 strains highly resemble each other and cluster into subtype 3a even though derived from all three species and different counties (Table 1). Only three sequences (KY910799, KY910800 and KY910798) delivered from humans in 2016 cluster under subtype 3c, however they remain closely related to the other 2016 sequences and one H-HEV sequence from 2014.

**Table 1:** Results of HEV RNA testing of samples derived from humans, swine and wild boars according to County of origin; number of tested/positive samples (percentages and 95% CI values); typing of recorded genotype 3 strains with subgrouping (SB) results and year of appearance

	Human	Swine	Wild boar	Typing results (G3)	Year
County	RT-PCR	RT-PCR	RT-PCR	ST	
BB	1/0	25/2 (8.0%; 2.22–24.97%)	n.d.	–	2010
BP	6/5	133/8 (6.0%; 3.1–11.4%)	143/34 (23.8%; 17.6–31.4%)	a, e, f	2012, 2013, 2016
DN	8/3	n.d.	13/0	a, f	2010, 2017
I	3/0	2/0	n.d.	–	–
K	3/0	58/0	72/1 (1.4%; 1.3–7.5%)		2016
KK	n.d.	31/3 (9.7%; 3.4–24.9%)	36/0	e	2010
KZ	2/1	32/0	18/0	a	2012
M	n.d.	22/2 (9.5%; 2.7–28.9%)	70/2 (2.9%; 0.8–9.8%)	c	2013, 2016
OB	8/0	383/72 (18.8%; 15.2–23.0%)	84/0	a	2013
PS	1/0	38/0	42/0	–	–
PG	4/1	n.d.	1/0	a	2015
SM	3/0	157/55 (37.4%; 30.0–45.5%)	137/31 (22.6%; 15.8–29.5%)	a	2010, 2016
SD	1/0	22/0	n.d.	–	–
SK	3/0	2/1	n.d.	–	–
VP	1/0	247/38 (15.4%; 11.4–20.4%)	30/9 (30.0%; 16.7–47.9%)	e	2010, 2016
VS	0/0	165/34 (20.6%; 15.1–27.4%)	54/6 (11,1%; 5.2–22.2%)	a, c	2010, 2013, 2016,
ZAD	1/0	n.d.	n.d.	–	–
ZG	128/16 (12.5%; 7.8–19.3%)	102/9 (8.8%; 4.7–15.9%)	21/0	a, c, e, f	2012, 2013, 2014, 2015, 2016, 2017
TOTAL:	174/26 (14.9%; 10.4–20.1%)	1419/216 (15.2; 13.5–17.2%)	720/83 (11.5%; 9.4–14.1%)	–	–

*n.d. = no data

Figure 2 shows the results of sequence analysis of representative HEV RNA sequences, where one sequence may present a group of identical ones according to host, year of appearance and geographical origin. The detection of a particular subgroup according to the host, county, year of recognition is shown in Table 1.

## Discussion

On the basis of genetic analysis carried out in this study, we can confirm that HEV strains derived during 2010–2017 in Croatia are closely related regardless of their geographical origin or host. Our findings indicate interspecies transmission of HEV and/or mutual sources of infection based on the high genetic identity of strains recovered from humans, swine and wild boar. The introduction of HEV infection into Croatia has not been entirely revealed within this study, however due to the result of genetic analyses from 2010 to 2012, we may presume that the virus was introduced by trade or by direct or indirect contact of swine with infected swine and wild boar from Europe. Even though the analyzed sequences rarely indicate a geographical connection, a statistically higher incidence of HEV RNA positive findings in all three investigated species in Northern Croatia where most of the swine farms are located and where the natural habitat of wild boars is recorded, shows that a high density of swine and wild boars increases the risk of viral spread.

HEV RNA was detected in 26 (14.94%) human patients that were IgM or IgM/IgG positive indicating recent infection, whereas IgG positive anti-HEV antibody sera remained HEV RNA negative, as previously described [28]. One positive archive human sera sample from 2010 that remained unrecognized until our study confirms that the introduction of HEV infection into Croatia appeared earlier than the reported case in 2012.

In tested swine, S-HEV was found in nine of the investigated counties with a prevalence of 15.2% (Table 1). Positive faecal samples were detected in six counties regardless of the breeding system, confirming viral shedding in detectable doses via faeces, possibly into the environment. Since farmers, especially within ecological management systems use swine faeces as manure, this finding may be a potential source of viral spread as previously reported [16, 29, 30]. The viral prevalence found in wild boars was 11.5% within the range reported in other European countries. Positive wild boars were detected in six investigated counties with the highest RNA prevalence found in counties with the highest density of wild boars (VP, BP and SM). In all the positive counties, swine with a high RNA prevalence were detected as well. Our results are comparable to other reported European findings [4].

H-HEV, S-HEV and WB-HEV strains from Croatia show to be genetically highly related members of *Orthohepevirus* A genotype 3. However, differences among them were observed. The derived strains clustered into two general clusters (3abchij and 3efg) [13], and furthermore into four subtypes (3a, 3c, 3e, 3f). Strains identified as members of subtype 3a and 3c are predominant in Croatia and may be considered endemic (Table 1). Only in KK County subtype 3a was not identified probably due to a limited number of tested samples.

H-HEV strains from subtype 3a show to be closely related to strains derived from wild boars, especially strains from SM County indicating a mutual source of infection or interspecies transmission of HEV. The estimated number of wild boars (according to the hunting bag) in Croatia varies from 45,700 to 65,000 per year. Since big game hunting is an important activity in this area, direct or indirect contact between hunters and wild boars is inevitable. Consumption of uncooked wild boar meat may also be considered an additional risk for HEV interspecies transmission in Croatia since H-HEV strains derived during 2012 to 2017 are genetically identical with some WB-HEV strains from 2010, 2015, 2016 and 2017.

S-HEV strains of subtype 3a show a higher genetic identity with strains derived from swine from neighbouring Serbia (HM483381 and HM 483383 from 2010) suggesting possible transmission of HEV by indirect contact, since import of live swine from Serbia is banned, or a mutual source of infection. To understand this finding additional testing of probable sources, including water, must be conducted.

H-HEV strains from subtype 3c appeared in 2010 and 2012 and have shown to be genetically identical to a swine and wild boar strain from Germany (JN415697 from 2009) and a swine strain from The Netherlands (EF372555 from 2005). Members of the same subtype were also derived from swine in 2013 and 2014 and show to be closely related to a human and a swine strain from the Netherlands (JQ929092 from 2010, EF372542 from 2005). We can only speculate the possible routes of infection in both cases. Croatia imports swine and swine products from Western European countries, so this entry could be a result of trade. Likewise, due to classical swine fever outbreaks in 2007–2008, the export of live pigs from Croatia to EU countries was banned or as of 2017 restricted from some regions in Croatia, so it’s less probable that the infection spread from Croatia to EU.

Two genetically identical HEV strains of human and swine origin, both from ZG County showed to be members of subtype 3e even though, they appeared in two different time periods. In swine they were derived in 2010 and 2012, whereas in humans in 2013 and 2014 indicating multiple entries. We can presume that the source of infection in patients is consumption of previously contaminated food of swine origin or the infection could be travel related. However, the source of infection in swine remains unidentified, as well as the reason why HEV 3e spreading or its persistence in the swine population is not recorded. Likewise, there is a lack of information in regards to the differences in virulence, duration of viraemia, host adaptability, as well as viral shedding of different HEV subtypes which may influence the detection of HEV RNA. Therefore, further investigations should be conducted in regards to the infection dynamics of HEV subtypes.

Members of subtype 3f were derived only from two patients, one in 2010 and the other in 2012. They show to be genetically highly related to strains derived from swine from the Netherlands (EF372555 from 2005) and a more distant strain derived from a human from Spain (AF195064, collection date is not mentioned in NCBI). Interestingly, subtype 3f strains have not yet been identified in swine/wild boars from Croatia and have not been detected in humans since 2012 indicating that this HEV subtype was imported.

According to the HEV RNA prevalence, the majority of HEV infected patients originate from ZG County where the largest genetic diversity of HEV strains was found. Since only small swine farms are represented in ZG County, the swine to human transmission was probably realised through consumption of contaminated homemade swine products, or by direct contact. This would correspondent with the fact that seroprevalence rates in Croatia in asymptomatic subjects are significantly higher in residents of suburban and rural areas [25] with a high density of small swine breeding farms. VS County borders with Serbia and Hungary and has the highest number of HEV positive swine and wild boars in Croatia, therefore finding homologous HEV strains derived from swine and wild boars (HM483383 from 2010 from Serbia and EU718646 from 2005 from Hungary) from these neighbouring countries is not surprising. S-HEV and WB-HEV strains are also genetically highly related among themselves confirming transmission of the virus, probably from swine farms to wild boars via contamination of the environment. However, further investigations should be carried out to identify additional HEV sources and transmission routes for a better understanding of HEV transmissibility.

## Conclusions

In conclusion, since 2010 HEV is circulating among swine and wild boars in Croatia [21] and from 2012, and possibly even earlier, in humans [24, 25]. Our results confirm that swine and wild boars represent a public health risk as reservoirs of HEV since we confirmed the presence of identical strains of HEV in all three investigated species. However, according to our results, some strains detected in Croatia (subtypes 3a and 3c) are more likely to persist among swine, wild boars and humans, while other strains show a rather sporadic occurrence with tendency of disappearing. To understand these epidemiological differences, further investigation is needed.

## Additional file

Additional file 1: Accession numbers, origin, year of detection and HEV subtypes of sequences of hepatitis E virus RNA used in this study. (DOCX 23 kb)

## Abbreviations

95%CI: 95% confidence interval
HEV: hepatitis E virus
IgG: immunoglobulin G
IgM: immunoglobulin M; ORF: open reading frame
P: *p*-value in Chi-square test
PBS: phosphate buffered saline
USA: United States of America
*w*/*v*: weight/ volume concentration
