# Supplementary material for: Genetic diversity of hepatitis E virus (HEV) strains derived from humans, swine and wild boars in Croatia from 2010 to 2017
Source: BMC Infect Dis. 2019 Mar 19;19:269. doi: 10.1186/s12879-019-3906-6 (PMC6425696; doi:10.1186/s12879-019-3906-6)
Supplement: Supplementary file 1 — Accession numbers, origin, year of detection and HEV subtypes of sequences of hepatitis E virus RNA used in this study. (DOCX 23 kb) [file 12879_2019_3906_MOESM1_ESM.docx]

| **GenBank accession no.** | **Collection**  **date** (year) | **Host species** (Human-H; swine-S; Wild boar-WB) | **Isolation source** | **Country** | **County** | **G** |
| --- | --- | --- | --- | --- | --- | --- |
| KJ850446 | 2010 | S | serum | Croatia | VP | 3e |
| KJ850455 | 2010 | S | serum | Croatia | VS | 3a |
| KJ850447 | 2010 | S | serum | Croatia | KK | 3e |
| KJ850463 | 2010 | S | serum | Croatia | SM | 3a |
| KT583113 | 2013 | S | feces | Croatia | BP | 3a |
| KT583114 | 2013 | S | feces | Croatia | ZG | 3e |
| KT583115 | 2013 | S | feces | Croatia | M | 3c |
| KT583116 | 2013 | S | feces | Croatia | M | 3c |
| KT583117 | 2013 | S | feces | Croatia | M | 3a |
| KT583118 | 2013 | S | feces | Croatia | OB | 3a |
| KT583119 | 2013 | S | feces | Croatia | OB | 3a |
| KT583120 | 2013 | S | feces | Croatia | VS | 3c |
| KT583121 | 2013 | S | feces | Croatia | VS | 3c |
| KT583122 | 2013 | S | feces | Croatia | VS | 3c |
| KT583129 | 2010 | H | serum | Croatia | DN | 3f |
| KT583123 | 2012 | H | serum | Croatia | ZG | 3a |
| KT583124 | 2012 | H | serum | Croatia | ZG | 3c |
| KT583125 | 2012 | H | serum | Croatia | KZ | 3a |
| KT583130 | 2012 | H | serum | Croatia | BP | 3f |
| KT583126 | 2013 | H | serum | Croatia | BP | 3a |
| KT583127 | 2013 | H | serum | Croatia | ZG | 3e |
| KT583128 | 2013 | H | serum | Croatia | ZG | 3c |
| KY910803 | 2014 | H | serum | Croatia | ZG | 3a |
| KY910801 | 2014 | H | serum | Croatia | ZG | 3a |
| KY910806 | 2014 | H | serum | Croatia | ZG | 3c |
| KY910804 | 2015 | H | serum | Croatia | ZG | 3a |
| KY910805 | 2015 | H | serum | Croatia | PG | 3a |
| KY910797 | 2016 | H | serum | Croatia | ZG | 3c |
| KY910798 | 2016 | H | serum | Croatia | ZG | 3c |
| KY910799 | 2016 | H | serum | Croatia | ZG | 3c |
| KY910800 | 2016 | H | serum | Croatia | ZG | 3c |
| KY910802 | 2017 | H | serum | Croatia | DN | 3a |
| KY910807 | 2017 | H | serum | Croatia | DN | 3a |
| KY910808 | 2017 | H | serum | Croatia | ZG | 3a |
| KJ850465 | 2010 | WB | serum | Croatia | SM | 3a |
| KJ850449 | 2010 | WB | serum | Croatia | BP | 3e |
| KY910809 | 2016 | WB | serum | Croatia | VS | 3a |
| KY910810 | 2016 | WB | serum | Croatia | VP | 3a |
| KY910811 | 2016 | WB | serum | Croatia | VS | 3a |
| KY910812 | 2016 | WB | serum | Croatia | VS | 3a |
| KY910813 | 2016 | WB | serum | Croatia | K | 3a |
| KY910814 | 2016 | WB | serum | Croatia | VS | 3a |
| KY910815 | 2016 | WB | serum | Croatia | VS | 3a |
| KY910816 | 2016 | WB | serum | Croatia | VS | 3a |
| KY910817 | 2016 | WB | serum | Croatia | BP | 3a |
| KY910818 | 2016 | WB | serum | Croatia | SM | 3a |
| KY910819 | 2016 | WB | serum | Croatia | SM | 3a |
| KY910820 | 2016 | WB | serum | Croatia | M | 3a |
| KY910821 | 2016 | WB | serum | Croatia | VS | 3a |
| KY910822 | 2016 | WB | serum | Croatia | VS | 3a |
| KY910823 | 2016 | WB | serum | Croatia | VS | 3a |
| KY910824 | 2016 | WB | serum | Croatia | VS | 3a |
| KY910825 | 2016 | WB | serum | Croatia | VS | 3a |
| EU718646 | 2005 | WB | liver | Hungary |  | 3a |
| HM483383 | 2010 | S | feces | Serbia |  | 3a |
| HM483381 | 2010 | S | feces | Serbia |  | 3a |
| AY115488 | - | S | feces | Canada |  | 3j |
| JQ929092 | 2010 | H | serum | Netherlands |  | 3c |
| FJ705359 | 2006 | WB | liver | Germany |  | 3c |
| JN415697 | 2009 | S | liver | Germany |  | 3c |
| EF372542 | 2005 | S | feces | Netherlands |  | 3c |
| EF372555 | 2005 | S | feces | Netherlands |  | 3f |
| AF195064 | - | H | serum | Spain |  | 3f |
| AF336003 | 1999 | S | feces | Netherlands |  | 3f |
| AB108537 | 2000 | H | serum | China |  | 4 |
| AB573435 | 2009 | WB | serum | Japan |  | 5 |
| AB602441 | 2006 | WB | liver | Japan |  | 6 |
| KJ496143 | 2013 | Camel | feces | United Arab Emirates |  | 3f |
| AY535004 | - | Chicken | bile | United States |  | HEV B |
| M74506 | - | H | feces | Mexico |  | 2 |
| M73218 | - | H | feces | Burma |  | 1 |
| AB291962 | 2004 | H | serum | Japan |  | 3b |
| JN837481 | 2008 | H | serum | United States |  | 3a |
| JQ013794 | 2007 | H | seum | France |  | 3h |
| FJ998008 | 2007 | WB | bile | Germany |  | 3i |
| AF455784 | 1987-1989 | S | feces | Kyrgyzstan |  | 3g |
| KF922359 | 2009-2010 | H | serum | France |  | 3e |
| AF336013 | 1998-1999 | S | feces | Netherlands |  | 3c |
| AB850879 | 2012 | H | serum | Japan |  | 3f |
